# Heartland Virus Epidemiology, Vector Association, and Disease Potential

**DOI:** 10.3390/v10090498

**Published:** 2018-09-14

**Authors:** Aaron C. Brault, Harry M. Savage, Nisha K. Duggal, Rebecca J. Eisen, J. Erin Staples

**Affiliations:** 1 Division of Vector-Borne Diseases, USA Centers for Disease Control and Prevention, National Center for Emerging Zoonotic Infectious Diseases, 3156 Rampart Road, Fort Collins, CO 80521, USA; hms1@cdc.gov (H.M.S.); dyn2@cdc.gov (R.J.E.); auv1@cdc.gov (J.E.S.); 2 Department of Biomedical Sciences and Pathobiology, College of Veterinary Medicine, Virginia Polytechnic Institute and State University, 1981 Kraft Drive, Room 2033, Blacksburg, VA 24061, USA; nduggal@vt.edu

**Keywords:** Heartland virus, *Amblyomma americanum*, lone star tick, transmission, thrombocytopenia

## Abstract

First identified in two Missouri farmers exhibiting low white-blood-cell and platelet counts in 2009, Heartland virus (HRTV) is genetically closely related to severe fever with thrombocytopenia syndrome virus (SFTSV), a tick-borne phlebovirus producing similar symptoms in China, Korea, and Japan. Field isolations of HRTV from several life stages of unfed, host-seeking *Amblyomma americanum*, the lone star tick, implicated it as a putative vector capable of transstadial transmission. Laboratory vector competence assessments confirmed transstadial transmission of HRTV, demonstrated vertical infection, and showed co-feeding infection between *A. americanum*. A vertical infection rate of 33% from adult females to larvae in the laboratory was observed, while only one of 386 pools of molted nymphs (1930) reared from co-feeding larvae was positive for HRTV (maximum-likelihood estimate of infection rate = 0.52/1000). Over 35 human HRTV cases, all within the distribution range of *A. americanum*, have been documented. Serological testing of wildlife in areas near the index human cases, as well as in widely separated regions of the eastern United States where *A. americanum* occur, indicated many potential hosts such as raccoons and white-tailed deer. Attempts, however, to experimentally infect mice, rabbits, hamsters, chickens, raccoons, goats, and deer failed to produce detectable viremia. Immune-compromised mice and hamsters are the only susceptible models. Vertical infection augmented by co-feeding transmission could play a role in maintaining the virus in nature. A more complete assessment of the natural transmission cycle of HRTV coupled with serosurveys and enhanced HRTV disease surveillance are needed to better understand transmission dynamics and human health risks.

## 1. Introduction

In 2009, acute samples from two residents of northwestern Missouri were sent to the Centers for Disease Control and Prevention (CDC) to culture for suspected *Ehrlichia chaffeensis* infection. Both patients reported multiple tick exposures in the days prior to their illness. Presenting symptoms and signs for the patients were similar and consisted of fever, fatigue, anorexia (loss of appetite), diarrhea, leukopenia (low white-blood-cell count), and thrombocytopenia (low platelet count). Both patients developed more significant thrombocytopenia, as well as moderately elevated liver enzymes (transaminases), during hospitalization. Given the concern for ehrlichiosis, both received doxycycline; however, no significant improvement was noted. Although each sample did reveal cytopathic effects in culture, the characteristic morulae of *E. chaffeensis* were not observed. Subsequent work-up and evaluation of their clinical samples led to the discovery of Heartland virus (HRTV) [1]. 

Following 10–12 days of hospitalization, both patients were released home. One patient completely recovered within one month of his hospitalization. The other patient returned to normal activities but reported fatigue and headaches two years later. The clinical features of these cases were noted to be similar to illness caused by the only other tick-borne phlebovirus known to cause human disease, namely, severe fever with thrombocytopenia syndrome virus (SFTSV; *Phenuviridae*; *Phlebovirus*) [2,3,4].

## 2. Virology, Epidemiology, and Clinical Disease

### 2.1. Virology

**Figure 1:**
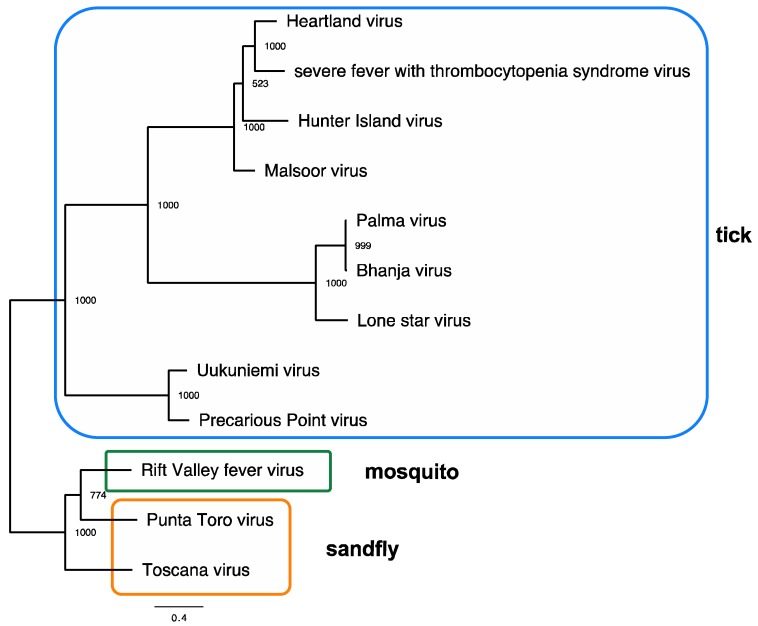
Maximum-likelihood tree showing genetic relationships among the tick-borne, mosquito-borne, and sand-fly-borne phleboviruses. A maximum-likelihood phylogeny based on amino-acid sequences from the large (L) segment from representative strains was generated in PhyML using amino-acid sequences. Bootstraps are indicated on nodes, and tick, mosquito, and sand-fly vector associations are indicated by colored boxes. The scale bar represents the mean amino-acid substitutions per site. Accession numbers: Uukuniemi virus D10759; Precarious Point virus HM566181; Toscana virus FJ153281; Rift Valley fever virus JQ068144; Punta Toro virus KR912212; Lone Star virus NC 021242; Palma virus JQ956379; Bhanja virus JQ956376; Hunter Island virus KF848980; Malsoor virus KF186497; Heartland virus JX005846; severe fever with thrombocytopenia syndrome virus HM745930.

HRTV (family *Phenuviridae*; genus *Phlebovirus*) has a genome comprising a segmented single-stranded negative-sense RNA genome encoded on small (S), medium (M), and large (L) segments [5]. Nucleocapsid (N) protein and a nonstructural (NSs) protein sequences are found on the S segment. The M segment encodes the structural glycoproteins, Gn and Gc, that serve to interact with host cellular receptors and serve as the primary targets of neutralizing immune responses. Finally, an RNA-dependent RNA polymerase involved in direct transcription of the viral genome is encoded on the L segment [6]. The phlebovirus serocomplex is a group of serologically cross-reactive viruses that phylogenetically associate with the arthropod vector associated with their transmission. Viruses within this group are transmitted by mosquitoes (such as the Rift Valley Fever virus; RVFV) or sand flies (such as the Toscana virus) in addition to ticks. Among the tick-borne phleboviruses, at least three groups are readily discernable: Uukuniemi, Bhanja, and SFTSV groups. Heartland viral sequences of the S, M, and L segments all segregate into the SFTSV clade, while other previously described North American phleboviruses, Lone Star virus (LSV) and Sunday Canyon virus (SCV), segregate with the Bhanja virus [7,8]. Additional tick-borne phleboviruses that segregate into the SFTSV clade are the Malsoor virus isolated from bats in 2010 from India, and the Albatross Island/Hunter Island virus isolated from *Ixodes* spp. ticks and moribund sea birds in Australia (Figure 1) [9,10,11].

Immune sera generated in mice against other North American phleboviruses such as LSV and SCV demonstrate at least four-fold differences in neutralizing capacity against HRTV. Additionally, HRTV human sera similarly exhibited a four-fold or lower titer against these heterologous phleboviruses than against homologous HRTV. Together, these data support the antigenic distinctness of these phleboviruses in North America.

The primary immune response measured against HRTV in animal studies is against the nucleocapsid (N), a viral protein that is not projected at the surface of the virion, and thus, immune responses against this protein are non-neutralizing [12,13]. Alternative phleboviruses, such as RVFV and Toscana virus, similarly demonstrate their N proteins to be immunodominant in experimentally inoculated animals, and, given that antibody responses generated against this protein are incapable of neutralizing free virus, it is suggested that this could serve as an immunological decoy for the hosts’ immune response [14,15,16,17,18]. The N protein is the most highly expressed viral protein detectable from phleboviral infected cells, and this abundance of viral protein undoubtedly is the basis for its immunodominance [18,19,20]. Nevertheless, evidence for some neutralizing responses to phlebovirus structural proteins was observed. A human monoclonal antibody capable of in vitro neutralization of SFTSV was generated [21].

The immunodominance of the N protein is also observed with other bunyaviruses, including tick-borne nairoviruses, for which antiviral T-cell-mediated responses were measured in vaccinated individuals [22]. Elevated levels of proinflammatory cytokines were measured in interferon-receptor-deficient mice infected with the nairovirus, Crimean–Congo hemorrhagic fever virus (CCHFV), that is associated with coagulopathy [23]. The NSs protein, implicated with antagonizing the host innate immune responses in orthobunyaviral infections [24], was shown to enhance replication of phleboviral RVFV minigenomes in mammalian cells [25]. This same viral protein was associated with viral clearance through suppression of the innate immune response in RVFV mouse models [26] and in experimentally infected ruminants [27]. 

### 2.2. Surveillance and Testing for HRTV Disease

Following the discovery of HRTV, the CDC began working with state and local partners to identify additional cases in order to describe the epidemiology, clinical symptoms and signs, and outcome [28]. In 2012, a prospective study protocol was implemented in selected Missouri hospitals to identify additional cases of HRTV, and, in 2013, CDC launched a national protocol to identify HRTV disease cases in other parts of the United States by offering diagnostic testing for patients with a clinically compatible illness. Both the Missouri and national protocols required patients to have a recent febrile illness (≥38 °C) with documented leukopenia (white-blood-cell (WBC) count of <4500 cells/µL) and thrombocytopenia (platelet count of <150,000 cells/mL). Individuals with known non-infectious etiologies (e.g., cancer or chemotherapy) or diagnosed with another infectious disease that may result in these clinical findings were excluded. The inclusion criteria were selected after reviewing the clinical signs and symptoms in the initial two cases and aimed to provide some specificity to persons who would be tested with newly developed diagnostic assays. The Missouri protocol stopped enrolment in 2015, and the national protocol concluded in 2017. Analysis and description of the results from those protocols, as well as the results from a serosurvey performed in northwest Missouri, are currently being finalized and are not available for inclusion herein.

The initial tests for identification of HRTV infection in humans included a molecular test (RT-PCR) to detect viral RNA, and a plaque reduction neutralization test (PRNT) to detect HRTV-neutralizing antibodies. However, the CDC also recently developed immunoglobulin M (IgM) and immunoglobulin G (IgG) microsphere immunoassays (MIAs).

Molecular testing for HRTV is currently available through a few state health departments and clinical institutions. Molecular and serologic testing is available at CDC Fort Collins, Colorado as part of their reference diagnostic, Clinical Laboratory Improvement Amendments (CLIA)-approved laboratory testing. HRTV is not currently a notifiable disease; however, the CDC asks that states report possible cases of HRTV disease on a voluntary basis. 

### 2.3. Epidemiology of HRTV

**Figure 2:**
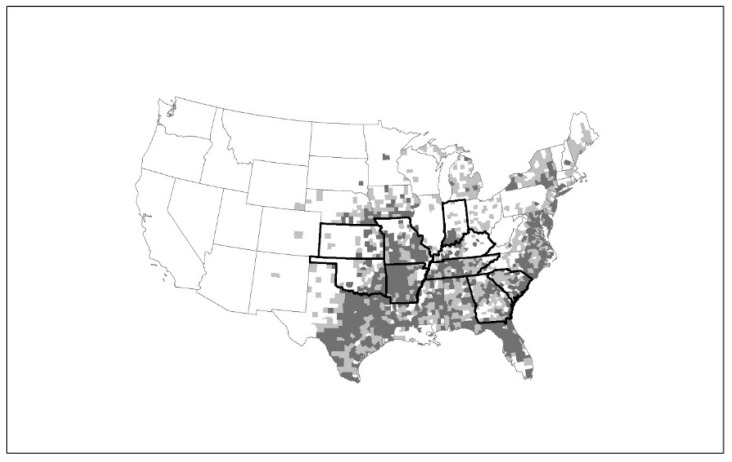
Heartland virus (HRTV) disease cases by state of residence and distribution of *Amblyomma americanum*. Map of the United States indicating states (Kansas, Oklahoma, Arkansas, Missouri, Tennessee, Kentucky, Indiana, Georgia, and South Carolina) with confirmed human HRTV cases (dark outline), as well as the county distribution of *A. americanum* representing established (dark-gray counties) and reported populations (light-gray counties) based on Reference [35].

**Figure 3:**
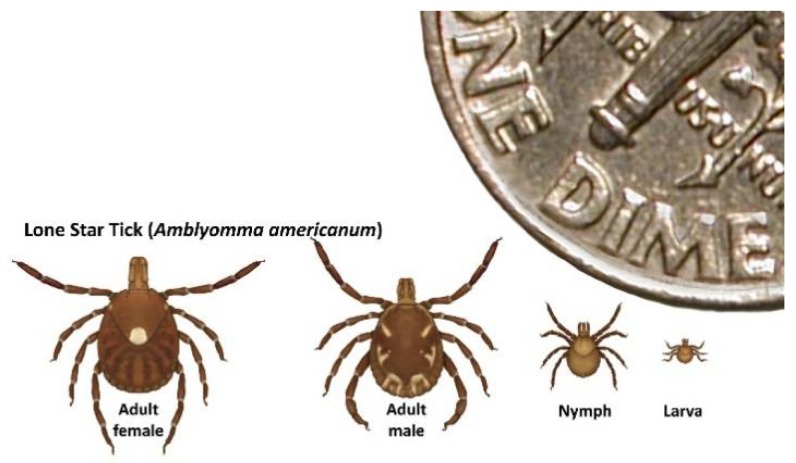
Life stages of *Amblyomma americanum*. *A. americanum* is implicated as a probable vector of HRTV in North America based on repeated field isolations, vector competence, and catholic host-feeding behavior. White-tailed deer serve as hosts for all three *A. americanum* [36] life stages, but all stages can feed on humans. Adults feed principally on middle-to-large-sized mammals, while larvae and nymphs typically feed on ground-associated birds, middle-sized mammals, and occasionally, on small mammalian hosts.

From July 2012–June 2018, over 35 disease cases were identified from states in the midwestern and southern United States [29] (Figure 2). The current distribution of HRTV disease cases mirrors closely the distribution of the lone star tick, *Amblyomma americanum* (Figure 2)*,* and most case-patients report either finding a tick attached to, or crawling on, their body in the two weeks prior to illness onset [28,30] (Figure 3). Several states with *A. americanum* have not yet identified HRTV disease cases. Although this might be due to differences in the geographic distribution of the agent, it also could be due, in part, to differences in enrolment and testing of residents of their states for the disease. Currently, no other mode of transmission is identified for HRTV. However, since some individuals with HRTV disease have high levels of virus in their blood, unprotected exposure to their blood could represent an infectious risk, as was reported with SFTSV [31,32,33,34].

For all cases identified to date, illness onset ranged from April to September with the majority developing symptoms in June. The median age of identified cases is 66 years (range: 29–80 years). Although, all 10 case-patients reported in the literature to date are males, females were found with the disease (CDC unpublished).

### 2.4. Clinical Disease

Clinical signs and symptoms are reported in the literature for the original two cases, two fatal cases, and six other cases identified through the established protocols [1,28,37,38,39]. All ten patients presented or had a history of acute onset of fever. Additional symptoms reported by the majority of cases included headache, fatigue, nausea, myalgia (muscle pain), arthralgia (joint pain), and weakness. Rash is typically not reported, though some case-patients reported a local rash that was often associated with their original tick bite. Several patients were reported to be confused or have frank mental-status changes. However, the two individuals who underwent lumbar punctures had no evidence of acute inflammation in their cerebrospinal fluid (CSF; WBC count of <5/µL), which would be seen with an infection of the brain (encephalitis) or meninges (meningitis).

All individuals with acute HRTV disease had thrombocytopenia at presentation; nine also had leukopenia. The case without leukopenia was noted to have leukocytosis (elevated white-blood-cell count) when measured later in their hospital course. At least one additional case with initial leukopenia also developed leukocytosis later in the second week of their illness [39]. Anemia (low red-blood-cell count), however, is typically not reported. For all cases where liver function tests were measured, all had elevated levels of enzymes, aspartate aminotransferase (AST) and alanine aminotransferase (ALT), typically peaking later in the second week of illness, and AST levels at least 2–3 times higher than ALT levels. Other noted laboratory abnormalities, particularly in more severely ill patients, include hyponatremia (low sodium blood levels), elevated bilirubin, increased creatine kinase, elevated lactate dehydrogenase, and markedly increased ferritin. For the cases with increased ferritin, one was documented to meet the diagnostic criteria for hemophagocytic lymphohistiocytosis (HLH; excess of activated immune cells) and the other was noted to have hemophagocytosis (phagocytosis of erythrocytes/lymphocytes) in their bone marrow, with granular immunostaining of viral antigens in the mononuclear cells [37,38]. One additional HRTV fatal case did not have ferritin measured, but had hemophagocytosis seen in a lymph node on autopsy. HLH and hemophagocytosis were also found to be associated with SFTS virus infection [40,41]. 

Of the ten documented HRTV disease cases, eight were hospitalized. Seven cases recovered and three died. All three deaths were in adult males aged >60 years. Two of the three fatal cases had multiple underlying medical co-morbidities, while the third was noted to have a history of intracerebral hemorrhage, melanoma, and hypertension. For the fatal cases, all progressed to develop acute renal/kidney injury and failure, respiratory failure, and hypotension (low blood pressure) consistent with sepsis, and died two to three weeks after their acute illness onset of multisystem organ failure. It is likely that the case–fatality ratio for HRTV disease is lower than what is currently documented, as there was clear case ascertainment bias with physicians requesting testing more often for severe cases without a clear diagnosis. By comparison, a case–fatality ratio of 6–17% was described for individuals identified with SFTSV infection in China, with the more severe clinical manifestation and poorer outcomes associated with older individuals [42]. 

The clinical symptoms, as well as the clinical signs of leukopenia and thrombocytopenia, seen with HRTV disease are very similar to those seen with ehrlichiosis, and they cannot be used to distinguish these two diseases which have overlapping geographic ranges. Clinical improvement within 48–72 h of starting appropriate antibiotics for ehrlichiosis (i.e., doxycycline) versus no substantial improvement of signs and symptoms after starting doxycycline could potentially be used to differentiate the most likely etiology.

### 2.5. Clinical Management

There are no known antiviral therapies available to treat HRTV infection, and management for acute disease is primarily supportive care. Antipyretics and analgesics can be used to reduce the fever and pain. More severe cases might need ventilator support, vasopressors, blood products, or dialysis. For the SFTS virus, a number of therapeutics (e.g., ribavirin, steroids) were administered in attempts to lower disease morbidity and mortality, but none were found to be effective in placebo control trials or for most patients [43,44,45].

## 3. Ecological Assessments of HRTV Transmission

### 3.1. Detection of HRTV in Field-Collected Ticks

HRTV was detected in field-collected host-seeking nymphal and adult *A. americanum* (L.) ticks collected in two states, Missouri and Kansas, where intensive field collections and tick testing were conducted [46,47,48]. Initial field studies conducted during 2012 in northwestern Missouri at 12 sites, including the residences of the two, original case-patients, produced 10 HRTV-positive tick pools, all composed of unfed, host-seeking nymphs [1,46]. Nine of the 10 positive pools (90%) were collected on a farm owned by one of the original case-patients (site 1), with one virus-positive pool collected on a nearby state conservation area [46]. Collection of host-seeking *A. americanum* in the following year (2013) in northwestern Missouri at six sites, including the residences of the two original case-patients plus three new case-patients identified by a hospital-based surveillance system, resulted in the detection of 60 virus-positive *A. americanum* pools [28,47]. Fifty-three HRTV-positive pools were composed of nymphs, one female and six male pools. In 2013, HRTV-positive tick pools were detected at four of the five properties owned by case-patients, including the original case-patient residence (site 1), plus the nearby state conservation area that yielded virus-positive ticks in 2012 [47]. Detection of HRTV at the same site, including human-case residences, in multiple years indicates that the virus likely overwinters and persists within environmentally suitable locations [47]. 

Infection rates for HRTV in *A. americanum* were shown to be highly variable among collection sites, at the same site in different years, and among life stages. However, an estimate of infection rate is important for accurate public-health messaging and improved understanding of virus transmission dynamics. The infection rate for HRTV in nymphs, the most commonly collected stage, from all sites in Missouri during the 2012 season was 1.4/1000, or one infected nymph of 719 nymphs tested [46]. Similarly, the infection rate for HRTV in nymphs from all sites in Missouri during the 2013 season was 1.8/1000, or one infected nymph of 559 nymphs tested [47]. Combining data on nymphs from the 2012–2013 Missouri collections produces an overall infection rate of 1.7/1000, or one in 585 *A. americanum* nymphs. Although infection rates are highly variable among species of ticks and viruses, similarly low rates of SFTSV infection were observed in field-collected *Haemaphysalis* spp. ticks in China [49].

To date, the virus remains undetected in field-collected, unfed larvae, and testing of 45,760 larvae collected in Missouri in 2012 failed to yield a virus-positive pool [46]. However, experimental transmission studies document that vertical transmission of HRTV from infected female to larvae may occur, and HRTV likely occurs in nature in all three life stages [50]. HRTV was detected in field-collected host-seeking nymphs in April, June, July, and August, and in host-seeking adult ticks in April and June [46,47,48]. Detection of HRTV in host-seeking nymphs and adult ticks in April suggests that the virus overwinters in these stages in Missouri and geographically similar areas. 

SFTSV is thought to be transstadially passaged from the larval to the nymphal stage in ticks (*Haemaphysalis longicornis*) and transmitted to humans by blood-feeding nymphs [49,51]. Furthermore, larval ticks are thought to be exposed to SFTSV by feeding principally on viremic domestic farm animals, such as goats. Infection and subsequent transstadial transmission to nymphal stages is postulated, allowing for horizontal transmission of the agent to a host species at the subsequent life stage with the tick’s next blood feed. Tick exposures reported for the initial human cases [1], the identification of nymphal and adult ticks infected with HRTV [46,47], and the aforementioned SFSV tick-associated transmission [52] and the tick-associated transmission of the other viruses in the SFTSV clade to which HRTV is phylogenetically placed (Figure 1) all contributed to HRTV being considered as an agent presumptively transmitted by ticks. Subsequent laboratory vector competence work with lone star ticks (see below) corroborated this initial assertion. This vector is known to be a catholic feeder, feeding on numerous medium-to-large vertebrate hosts [36,53]. 

### 3.2. Experimental Vector Competence Studies with HRTV and A. americanum

Detection of HRTV in field-collected nymphs and adult ticks highlighted the need for laboratory-based vector competence studies with HRTV and *A. americanum*. *Amblyomma americanum* ticks were obtained from a colony initiated in 1999 from specimens collected in Atlanta, Georgia (GA), and were maintained by the Rickettsial Zoonoses Branch, Centers for Disease Control and Prevention in Atlanta, GA. As an animal model for HRTV was not available, the immersion technique was employed to infect larvae with HRTV [50,54]. 

After immersion in concentrated HRTV (6.3 log_10_ plaque-forming units (PFU)/mL) solution, larvae were placed in a back bag on a New Zealand white rabbit and allowed to blood feed [50,55]. Engorged larvae were collected, washed, and tubed, and were allowed to molt to the nymphal stage. A subsample of 80 nymphs were individually tested for HRTV. Thirty-one nymphs (39%) were RT-PCR-positive and 28 (35%) were HRTV-positive via plaque assay, demonstrating transstadial transmission of viable virus from the larval to nymphal stages. 

In addition to possible vertical infection from their mothers, ticks may also acquire HRTV from viremic hosts or by co-feeding (see below) with infected ticks, and transstadially transmit the virus to the subsequent stage [50]. An orthomyxovirus, Thogoto virus (THOV), was reported to be transmitted between ticks while co-feeding [56]. To assess HRTV infection by co-feeding, 175 potentially infected *A. americanum* nymphs (virus donors) produced as described above were placed in a rabbit bag with larvae from a clean-colony egg batch (virus recipients). The tick bag was checked daily and engorged ticks that dropped off were cleaned and placed in tubes [57]. At 8–14 days post drop off, potential donor nymphs were individually tested for HRTV. Fifty-two (37%) of 142 surviving nymphs were RT-PCR-positive and 44 (31%) were virus-positive via plaque assay. The virus was not detected in a subsample of 100 engorged larvae. However, testing of 1930 nymphs that molted from the remaining engorged larvae in 386 pools of five nymphs resulted in the detection of one HRTV-positive pool both via RT-PCR and plaque assay and a maximum-likelihood estimate of the infection rate of 0.52/1000. Transmission of HRTV to larvae by infected nymphs was demonstrated, but the low rate suggests co-feeding among these two stages may not be an effective maintenance mechanism. Whether natural co-feeding behaviors of ticks in the field or life-stage dependency (i.e., alternatively from larvae to nymph, adult to nymph, or nymph to adult) could facilitate greater efficiency of co-feeding infections remains to be seen. Nevertheless, the HRTV infection rate in potential donor nymphs used in the co-feeding experiment was not significantly higher than that of the subsample of 80 unfed nymphs from the immersion experiment not exposed to infected co-feeding nymphs, supporting a lack of infection by co-feeding among nymphs.

Another group of nymphs that molted from immersed larvae was allowed to feed on a rabbit to produce transstadially infected adults. Forty pairs of adults were allowed to feed on a rabbit, 20 pairs in each of two back bags. Thirty-nine fed and unfed females and 36 males were recovered and individually tubed. Females were allowed to oviposit and were then placed in clean labeled tubes and frozen at −80 °C. Twelve of 36 (33%) male ticks were HRTV-positive via RT-PCR and 10 (28%) were positive for viable virus via plaque assay. Twenty-one of 39 (54%) female ticks were RT-PCR-positive for HRTV, and 17 (44%) adult females were virus-positive via plaque assay. Infection rates between male and females were not significantly different. Fifteen of 21 (71%) infected females oviposited. To assess vertical transmission from infected females to their progeny, larvae from each infected female that oviposited were tested in pools of ≤100. HRTV RNA was detected in larvae from five of 15 (33%) infected females. All larval pools from four of the five infected females were RT-PCR-positive for HRTV, while 40% of pools were HTRV-positive for the fifth female that vertically transmitted virus to her progeny. Overall, 23% (28/122) of larvae pools from infected females were RT-PCR-positive, with 20% (24/122) yielding live virus, indicating that vertical transmission could be propagated under laboratory conditions.

Godsey et al. (2016) demonstrated transstadial transmission of HRTV from larvae to nymphs, and from nymphs to adults in the tick *A. americanum*. Infection of larvae from infected nymphs by co-feeding was also demonstrated, but at very low rates. In contrast, vertical transmission may be an important maintenance mechanism, as vertical transmission of HRTV from infected female to larval progeny occurred in 33% of infected females, and at very high rates in females that vertically transmitted the virus [50].

**Figure 4:**
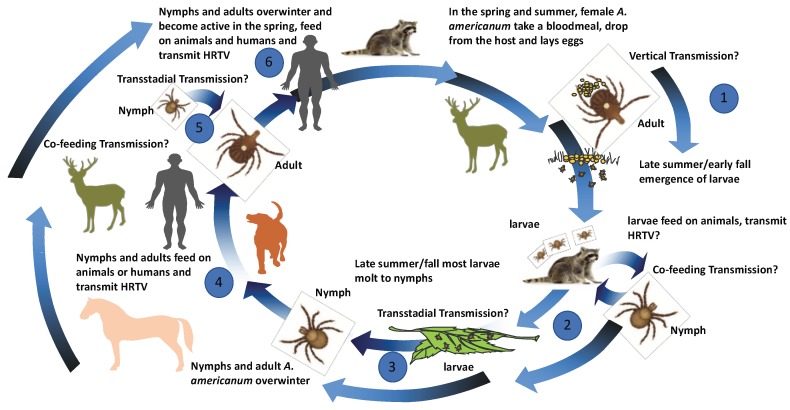
Proposed transmission model for the Heartland virus. Proposed transmission cycle for HRTV between multiple life stages of *A. americanum* and various vertebrate hosts. (1) Adult *A. americanum* feed on white-tailed deer and become infected or were previously infected and possibly vertically pass HRTV infections to larvae. (2) Larvae feed on middle-sized mammalian hosts and possibly transmit HRTV or become infected while co-feeding on hosts with HRTV larvae or ticks of different life stages. (3) Larvae possibly transstadially passage HRTV to nymphal stage. (4) Nymphs and adult infected transstadially or by co-feeding possibly transmit HRTV to humans, middle- or large-sized mammalian hosts. (5) Co-feeding or transstadial transmission could occur from nymphal to adult stage. (6) Overwintering of infected nymphal or adult stages could occur with possible transmission in the spring.

Based on the published data on the transmission capacity of *A. americanum* for HRTV, the experimental results of co-feeding and vertical transmission experiments, as well as the host competence work summarized above, a proposed transmission cycle is schematically displayed (Figure 4). 

### 3.3. Phleboviral/HRTV-Neutralizing Antibodies in Field-Collected Animals

Serum samples from domestic and wild animals collected in the areas adjacent to the sites of the initial human HRTV cases [58], as well as throughout the United States in areas with known distributions of *A. americanum*, demonstrated phlebovirus-neutralizing antibodies [59,60]. In areas adjacent to the initial human cases, phlebovirus-neutralizing antibodies were identified in white-tailed deer, horses, raccoons, opossums, and dogs. 

White-tailed deer showed the highest neutralizing-antibody rate (64%) followed by raccoons (55–68%), horses (22%), and dogs (8%). However, more specific HRTV-neutralizing antibodies were detected in higher rates for raccoons (43%) and horses (17%) than white-tailed deer (14%). Despite being heavily parasitized by *A. americanum*, opossums demonstrated only a 4% HRTV neutralization rate. Larval, nymphal, and adult *A. americanum* were implicated with avian blood-feeding [53,61]; however, none of the 132 processed bird serum samples, representing 26 different avian species, demonstrated any detectable neutralizing antibodies. White-tailed deer and raccoons, two species that were found to have the highest phlebovirus seropositivity rates, were documented to serve as hosts for *A. americanum* life stages [36]. Domestic animal sera samples taken from animals in close proximity to human SFTSV cases in China identified 95% seropositivity in goats and owning a goat was identified as a potential risk factor for SFTSV infection [51,62,63]. One case of SFTSV infection was associated with exposure via bite from an infected domestic cat [64] and cat ownership was identified as an independent risk factor associated with SFTSV infections in China [65]. Furthermore, 18% of feral cats in Seoul, South Korea were identified to be positive for SFTSV RNA [66]. Only two sera samples from domesticated cats were assayed for neutralizing antibodies to HRTV in northwestern Missouri with both negative via PRNT assay. Nevertheless, the association with infection and transmission risk of SFTSV indicates that cat exposure rates to HRTV might warrant further evaluation with larger numbers. 

Serosurveys serve exclusively to demonstrate field exposure rates to the viral antigen and do not per se provide any direct evidence that the vertebrate host serves as an amplification host; however, results of these studies can be quite informative regarding the geographic distribution of HRTV and the establishment of sentinel tracking systems. A large animal survey from across the central and eastern United States demonstrated HRTV-neutralizing antibodies in deer, raccoons, coyotes, and moose from thirteen states [59]. This field serosurvey indicated that a variety of domestic and wild mammals were exposed to HRTV or a related phlebovirus in areas that are largely coincident with the known geographic distribution of *A. americanum*. Phlebovirus-seropositive animals identified in Minnesota in areas not known to harbor populations of *A. americanum* were screened with an SFTSV antigen [60]. This antigen could result in cross-reactivity with sera from exposure to alternative North American phleboviruses that could utilize alternative tick vectors. The same could be indicative, for instance, for moose sera that were phlebovirus-positive from Maine, an area beyond the currently described geographic distribution for *A. americanum* [59]; however, the geographic range of *A. americanum* expanded dramatically in recent years and the areas of the index cases were not known to have established tick populations prior to the identification of the two human index HRTV cases [67]. 

## 4. Host Competence and Pathogenesis Assessments

### Animal Model Assessments

Experimental animal infection studies with HRTV were undertaken to (i) model human disease processes, (ii) assess the potential of a vertebrate host to serve as an amplifying host involved in the natural transmission of the agent in the field, and (iii) serve as a source for the establishment of a tick–vertebrate laboratory transmission model for assessing of vector competence. Similar to SFTSV, only limited experimental animal infection studies were performed with HRTV. Based on the high neutralizing-antibody rates and the wide geographic range of northern raccoons within the United States [59] and in areas adjacent to the index human HRTV cases [58], six field-collected, HRTV-seronegative raccoons were used for experimental inoculation with HRTV. Despite seroconversion after primary and secondary viral challenge with HRTV, none of the raccoons demonstrated any clinical signs of disease or detectable viremia [12]. A natural experimental study with naive goats was similarly performed with SFTSV in which goats developed detectable viremia after field-tick exposure based on the detection of high antibody-positivity rates in proximal areas to human disease cases [63,68]. Unlike goats exposed to SFTSV through tick bite, needle-inoculated goats with HRTV failed to develop any detectable viremia, but did seroconvert. Similarly, chickens, rabbits, and hamsters, although developing antibody responses to HRTV, also failed to develop detectable viremias following experimental needle inoculation [12]. Similar to the results obtained from experimental inoculation of field-captured raccoons, experimental needle inoculation of white-tailed deer fawns resulted in no detectable viremia or observable pathology with low-level immune responses measured [69]. These experimental inoculation studies with HRTV were all performed via needle inoculation. Given the results of field-exposed goats to SFTSV, as well as the well-characterized immune modulatory effects of tick salivary proteins, it is possible that long-term exposure of some of the vertebrate hosts to ticks could result in detectable viremia sufficient to infect ticks. A comparison of animal models that were utilized for HRTV and SFTSV is provided in Table 1. 

SFTSV was shown to replicate to detectable levels and demonstrate lesions in the spleens in experimentally inoculated immune-competent C57BL/6 mice [71]; however, HRTV experimental inoculation of this same mouse model failed to demonstrate either lesions or detectable viremia [12]. When outbred CD-1 mice were experimentally inoculated intracranially with either LSV or HRTV, only LSV-inoculated mice showed symptoms following inoculation, indicating that, in addition to being incapable of eliciting disease in immune-competent mice inoculated peripherally, HRTV also failed to demonstrate neurovirulence properties in immune-competent mice [12]. In contrast, when type I and type II interferon (IFN)-receptor-deficient mice (AG129) were inoculated via intraperitoneal route, a dose-dependent response was observed with a lethal dose 50% (LD_50_) of <10 plaque-forming units (PFU) of HRTV. Mice that were inoculated with 10^4^ PFU of HRTV developed viremias >8 log_10_ PFU/mL sera. The requirement for the use of an immune-compromised mouse complicated the development of an animal-tick HRTV infection model given the low feeding efficiency of *A. americanum* on mice. Mice at the higher (3–4 log_10_ PFU/mL sera) inoculation dose groups typically succumbed to infection within 5–6 days with mice receiving as little as 1 PFU succumbing at 8–11 days with lower viremias (<4 log_10_ PFU/mL sera). AG129 mice exhibited clinical signs that included mucopurulent ocular discharge, sick rodent posture, and hematochezia (non-digested blood in the rectum). Spleen and liver tissues demonstrated apoptotic debris and mononuclear cellular infiltrates. The HRTV antigen was readily observed in interstitial mononuclear cells of the kidney, Kupffer cells in the liver, and splenic mononuclear cells [12]. Although SFTSV experimentally inoculated immune-competent mice were found to undergo pathological processes equating to leukopenia and thrombocytopenia observed in humans, these manifestations were observed to be mild, and replication of SFTSV was limited to the spleen [71]. In contrast, similar to results observed with type I/II IFN receptor deficient mice inoculated with HRTV, type I interferon-deficient mice inoculated with SFTSV were demonstrated to be highly susceptible to lethal infection, with elevated viremias [71,74]. 

Experimental inoculation experiments with both SFTSV and HRTV showed the protective effect of the interferon response within mouse models. Similarly, virulence differences identified with the Punto Toro virus (PTV) strains, another phlebovirus that was shown to be virulent in immune-competent mice and hamsters, were associated with differential capacities of suppressing IFN-beta responses [78,79]. Hamsters deficient in interferon type-I signaling also demonstrated susceptibility to lethal infection with HRTV, serving as further evidence of the importance of the interferon response of HRTV in an alternative rodent model [75]. The affinity of phleboviruses for infection of mononuclear cells likely results in robust stimulation of interferon responses directed by infection of these cells. Studies are warranted to specifically assess the role of these cell populations for trafficking HRTV within infected hosts, and the direct and indirect role this tropism has on pathological development of the disease in humans (thrombocytopenia and leukopenia).

As stated earlier, the N protein of bunyaviruses elicits the strongest humoral response which does not result in neutralizing immunity, as evidenced by monoclonal antibodies generated from spleens taken from HRTV-inoculated AG129 mice which all targeted the nucleocapsid and were found to be non-neutralizing [13]. A full appreciation of both the modeling of human disease processes in animal models, as well as the assessment of vertebrate host species as potential amplification hosts, will require biologically relevant tick transmission experiments. The immunomodulatory effects that tick saliva may have on the induction of host innate immune response could be a critical factor for overall host competence to HRTV [80,81] or could mediate co-feeding infection efficiency. Tick transmissions of THOV and the Powassan virus were shown to potentiate elevated viremia production in animal models compared to animals administered virus via needle delivery, implicating salivary immune modulative factors such as the antagonism of the interferon response of the host by tick saliva [80,82].

## 5. Conclusions

In the ten years following the identification of HRTV in North America, considerable information on the viral agent, its vector transmission potential, and human disease risk was assembled. Nevertheless, there are considerable questions that remain to be answered. For instance, are vertebrate hosts that develop a viremia required for maintenance of HRTV between *A. americanum* ticks or can vertical transmission serve as the principal source of transmission augmented by horizontal co-feeding infections? Human serosurveys will be necessary in order to truly address the exposure risk and the symptomatic-to-asymptomatic infection rate experienced by humans. Dual infections of bacterial agents and SFTSV were described in ticks [83]. The role of dual infection of ticks with HRTV and bacterial agents (e.g., *Ehrlichia* species) also vectored by *A. americanum* on transmissibility is yet to be assessed, and the potential modulation of disease presentation in co-infected humans is yet to be investigated. The recent identification of multiple locations within the United States (New Jersey (overwintering), New York, Maryland, North Carolina, Pennsylvania, Virginia, and West Virginia), with infestations of the longhorned tick (*H. longicornis*), a proposed vector of SFTSV in Asia [49], confirmed by the United States Department of Agriculture (USDA) National Veterinary Services Laboratory, highlight the potential that imported ticks could either introduce exotic agents (SFTSV) or could serve as alternative vectors for endemic agents such as HRTV in North America [84]. It is very likely that HRTV circulated in North America for a considerable period of time in which human disease cases went unrecognized. Differences in host susceptibility (such as that observed between goats exposed to SFTSV and HRTV) could represent diversification of independently evolving phleboviruses in different environments over time. Although more information will assuredly be accrued in the coming years, the full extent of the human disease risk that HRTV presents is not currently established. 

## Figures and Tables

**Table 1:** Animal models assessed for Heartland virus (HRTV) and severe fever with thrombocytopenia syndrome virus (SFTSV).

Model	Heartland Virus	References	Severe Fever with Thrombocytopenia Virus	References
immunocompetent mice	C57bl/6 (i.p. inoculation): no viremia, morbidity/mortality, or clinical signs, seroconversion CD1 (i.c. inoculation): no morbidity/mortality	[12,70]	C57bl/6, BalB/C (i.v., i.m., i.p. inoculation): viremia, lesions, leukopenia through all routes and replication in spleen; thrombocytopenia (i.v. and i.m. routes); no significant morbidity or blood counts in BalB/C, but elicited liver/kidney lesions Newborn KM mice and rats (i.p. and i.c.): lethality and viremia and liver lesions, but adults were refractory CD1 newborn mice (i.c. inoculation): passage adaptation of viruses for consistent morbidity/mortality	[70,71,72,73]
immunodeficient mice	AG129 (Type I/II IFN^r^ KO; i.p. inoculation): high viremia and dose-dependent mortality	[12]	IFNAR^−/−^ (s.c. inoculation): detectable SFTSV RNA in serum with morbidity/mortality, reticular tropism in spleen	[70,73,74]
hamsters	Syrian: no detectable viremia or serum RNA, seroconversion; STAT2 KO (s.c. inoculation) showed viremia and morbidity/mortality	[12,75]	Syrian (i.v., i.m., i.p., i.c. inoculation): no significant morbidity or blood count alterations Newborn (i.c. inoculation) showed no morbidity/mortality and adult (i.p. inoculation) failed to generate detectable viremias and showed no outward signs of disease STAT2 KO (s.c. inoculation) showed viremia and morbidity/mortality	[7,73,76]
rabbits	New Zealand white (s.c. inoculation): no detectable viremia or serum RNA, low seroconversion rates; (tick feed): no detectable serum RNA with seroconversion	[12,50]		
raccoons	Field-collected (s.c. inoculation): no detectable viremia or serum RNA, low seroconversion	[12]		
ungulates	White-tailed deer (i.d. inoculation): no detectable viremia or clinical disease with modest seroconversion; goats (s.c. inoculation): no detectable viremia or serum RNA, low seroconversion	[12,69]	Goats (s.c. inoculation): transient viremia with seroconversion without outward signs of disease Goats (field-tick exposure): transient viremia with seroconversion without outward signs of disease	[68]
avian	chickens (s.c. inoculation): no detectable viremia or serum RNA, no detectable seroconversion	[12]		
non-human primates	Cynomolgus macaques (s.c. inoculated): no detectable viremia, no clinical signs or lesions	[70]	Rhesus macaques (i.m. inoculated): mild symptoms, viremia and seroconversion detected, thrombocytopenia, leukocytopenia, liver/kidney lesions Cynomolgus macaques (s.c. inoculated): no detectable viremia or lesions, thrombocytopenia in one animal	[70,77]
